# A mixed methods randomised control trial to evaluate the effectiveness of the journey to social inclusion – phase 2 intervention for chronically homeless adults: study protocol

**DOI:** 10.1186/s12889-019-6644-1

**Published:** 2019-03-22

**Authors:** Shannen Vallesi, Paul Flatau, Monica Thielking, Jessica L. Mackelprang, Kathryn M. Taylor, Louise La Sala, Jude Spiers, Lisa Wood, Karen Martin, Darja Kragt, Leanne Lester, Elizabeth Whittaker, Ryan J. Courtney

**Affiliations:** 1 Centre for Social Impact University of Western Australia, University of Western Australia, 35 Stirling Hwy, Crawley, Western Australia 6009 Australia; 2 School of Population and Global Health, University of Western Australia, Perth, Western Australia Australia; 3 School of Health Sciences, Swinburne University of Technology, Melbourne, Victoria Australia; 4 Faculty of Nursing, University of Alberta, Edmonton, Alberta Canada; 5 Health Promotion and Evaluation Unit, University of Western Australia, Perth, Western Australia Australia; 6 National Drug and Alcohol Research Centre, University of New South Wales, Sydney, New South Wales Australia; 7 NSW Ministry of Health, Sydney, New South Wales Australia

**Keywords:** Chronic homelessness, Housing, Health, Social inclusion, Australia, Longitudinal, Mixed methods, Protocol, Intensive case management, linked administrative data

## Abstract

**Background:**

Chronic homelessness is a problem characterised by longstanding inability to attain or maintain secure accommodation. Longitudinal research with homeless populations is challenging, and randomised controlled trials that evaluate the effectiveness of intensive, case management interventions aimed at improving housing and health-related outcomes for chronically homelessness people are scant. More research is needed to inform programmatic design and policy frameworks in this area. This study protocol details an evaluation of the Journey to Social Inclusion – Phase 2 program, an intervention designed to reduce homelessness and improve outcomes in chronically homeless adults.

**Methods/design:**

J2SI Phase 2 is a three-year, mixed methods, multi-site, RCT that enrolled 186 participants aged 25 to 50 years between 07 January 2016 and 30 September 2016 in Melbourne. The intervention group (*n* = 90 recruited) receives the J2SI Phase 2 program, a trauma-informed intervention that integrates intensive case management and service coordination; transition to housing and support to sustain tenancy; and support to build social connections, obtain employment and foster independence. The comparison group (*n* = 96 recruited) receives standard service provision. Prior to randomisation, participants completed a baseline survey. Follow-up surveys will be completed every six months for three years (six in total). In addition to self-report data on history of homelessness and housing, physical and mental health, substance use, quality of life, social connectedness and public service utilisation, linked administrative data on participants’ public services utilisation (e.g., hospitalisation, justice system) will be obtained for the three-year period pre- and post-randomisation. Semi-structured, qualitative interviews will be conducted with a randomly selected subset of participants and service providers at three time-points to explore changes in key outcome variables and to examine individual experiences with the intervention and standard service provision. An economic evaluation of the intervention and associated costs will also be undertaken.

**Discussion:**

Results of this trial will provide robust evidence on the effectiveness of J2SI Phase 2 compared to standard service provision. If the intervention demonstrates effectiveness in improving housing, health, quality-of-life, and other social outcomes, it may be considered for broader national and international dissemination to improve outcomes among chronically homeless adults.

**Trial registration:**

Australian New Zealand Clinical Trials Registry ACTRN12616000162415 (retrospectively registered 10-February-2016).

**Electronic supplementary material:**

The online version of this article (10.1186/s12889-019-6644-1) contains supplementary material, which is available to authorized users.

## Background

Whilst some people experience homelessness situationally for short-term periods [1, 2], others cycle in and out of homelessness episodically or experience prolonged periods of homelessness that may last for years. It is among this group—*the chronically homeless*—for whom intensive interventions are required to address complex health and psychosocial needs, and for whom costs are most pronounced [3].

Homelessness is a complex public health and social problem that is both a driver and a consequence of poor health, social exclusion and economic marginalisation [4–6]. Homelessness is often preceded by experiences of trauma [7–12]—becoming homeless in and of itself can be traumatic [13]—and being homeless increases vulnerability to violence and victimisation events [7, 10]. Fitzpatrick and colleagues contend that childhood trauma is often at the root of the most complex homeless trajectories [6]. In addition to the development of trauma-related psychopathology, such as posttraumatic stress disorder (PTSD), the experience of trauma can foster distrust of services and institutions [7], social dislocation and stigmatisation [14]. Growing recognition of the nexus between chronic homelessness and trauma has led to calls for the embedding of trauma-informed practice within services that work with people experiencing homelessness [10]. Additionally, homelessness is often associated with a myriad of health problems, including high rates of chronic diseases, intentional and unintentional injury, and mental health and substance use problems [15–23]. These difficulties lead to increased use of ambulance and acute hospital services, longer hospital admissions (in part exacerbated by a reluctance to discharge patients to non-accommodation states), more frequent readmissions, and more medical complications. These factors compound to create a significant cost burden to the healthcare system [3, 24–28]. Effective homelessness interventions may reduce healthcare use over time, thereby yielding significant savings for the healthcare system [29–34].

In recent years, there have been concerted efforts in several countries to end chronic homelessness through the introduction of programs that go beyond crisis support and the provision of shelter. Housing First programs, which emphasise a rapid transition into housing for those who are homeless irrespective of the ‘readiness’ for housing, for example, have been shown to be effective in supporting people to exit homelessness, to sustain housing tenancies, and to reduce use of publicly funded services (e.g., ambulance services or the criminal justice system) [35–40].

In November 2009, Sacred Heart Mission (SHM), a not-for-profit community agency that provides support for homeless people in Melbourne, Australia, launched a three-year pilot study of the Journey to Social Inclusion (J2SI) program. J2SI was designed to break the cycle of chronic homelessness through the provision of long-term, trauma-informed, intensive case management (i.e., low staff-to-client ratio), and employment-related and skills-based support. J2SI aimed to provide rapid access to permanent housing and to improve the health, social, and employment outcomes of participants who were homeless in the geographical area served directly by SHM. J2SI was evaluated in a randomised controlled trial (RCT) in which the intervention group (*n* = 40) received the J2SI program and the comparison group (n = 40) received standard service provision [41–45]. Eighty-one percent of participants completed the three-year pilot (retention dropped to 67% at four-year follow-up) and of these, 85% of J2SI Pilot participants were housed compared to 41% in the comparison group. Housing outcomes between the two groups narrowed at 12 months following the completion of the trial (i.e., end of support) as a result of a decrease in housing rates among the intervention group and an increase in the comparison group [45]. The authors attribute the intervention group decrease partly due to participant abandonment of property and non-payment of rent. However, they were unable to provide an explanation for the increase in housing for the comparison group [45]. It was also discovered that, compared to the comparison group, the J2SI group had lower levels of stress, anxiety and depression, and fewer emergency department (ED) presentations and days in hospital at the 48 month follow up compared to baseline [45].

Following the pilot study, the J2SI program was modified and refined in preparation for testing its scalability for broader dissemination. The modified program, J2SI Phase 2, is the subject of the present research. The target number of eligible participants was increased to 60 per group (compared to 40 in the pilot study), a higher case management load was implemented (1:6 rather than 1:4), and an enhanced trauma-informed care practice was embedded into the intervention (drawing on findings from a study on trauma among chronically homeless adults in Melbourne [10]). Recruitment was also extended beyond SHM to include other inner-city Melbourne-based services, which expanded the geographical catchment area of the study.

The research design for the J2SI Phase 2 project is a pragmatic, multi-site, RCT assessing the effectiveness of the J2SI Phase 2 intervention. This study protocol describes the evaluation methodology and contributes to the literature by demonstrating the implementation of a RCT methodology in a homelessness context at the intersection of health and social policy, in which multiple forms of data (quantitative and qualitative) and evaluation methodologies (impact evaluation and economic evaluation) are incorporated.

### Aims

The aim of this study is to determine whether the J2SI Phase 2 intervention is more effective than standard service provision in achieving improved housing, health, social and economic participation and well-being outcomes for adults experiencing chronic homelessness and to assess the cost (net of cost offset savings) of any positive differential outcomes achieved. Compared to standard service provision, it is hypothesised that the J2SI Phase 2 intervention will result in:

1. Faster access to and higher rates of permanent housing, and longer sustained tenancy. Permanent housing is defined as public, community and private housing with private amenities and formal tenure and/or ownership rights;
2. Improved mental health and well-being (i.e., lower rates of depression, anxiety, stress, loneliness and psychological distress, and improved general wellbeing, self-esteem and quality of life outcomes) and reduced high-risk use of drugs and alcohol;
3. Reduced health services utilisation (e.g., fewer ED presentations and hospital admissions) allowing for possible higher utilisation during Year 1 of the trial (due to previously unmet health needs being addressed);
4. Higher employment and enrolment in education and training, stronger social connections and participation in social activities, and lower police incidents and involvement in the justice system; and
5. Lower healthcare and justice related costs.

## Methods/design

This study is a three-year RCT that aims to test the effectiveness of J2SI Phase 2 relative to standard service provision. The methodology, conduct and reporting of this study are in accordance with the Consolidated Standards of Reporting Trials (CONSORT) and Good Clinical Practice (GCP) guidelines [46].

### Participants and recruitment

Recruitment began in December 2015 and occurred across three catchment areas in inner city Melbourne, Australia (centred on St Kilda, Fitzroy, and North Melbourne). Melbourne is a city of 4.5 million people in the state of Victoria, Australia. Posters advertising the study were displayed at each of the three participating agencies, all of which provide services to men and women experiencing homelessness. All individuals who presented for intake assessment at each of the sites were informed of the project. Interested persons were assessed for study eligibility by a key referring worker who subsequently set up an interview for those deemed eligible, during which written informed consent was obtained and the baseline survey completed. Data collection for the J2SI Phase 2 study commenced in January 2016 and concludes in December 2019.

Recruitment was designed to ensure a minimum of 60 active participants in either group at the end of the baseline survey period. The number of participants in the program was set by budget constraints and the need to ensure that the study had sufficient power to detect differences between groups. Based on attrition rates from the J2SI pilot study, researchers anticipated there would be some intervention group participants who would not engage with the J2SI Phase 2 program but that overall attrition would likely be greater in the comparison group as a result of infrequent contact (i.e., only for follow-up survey administration) and greater loss to follow-up. The study over-recruited, as we anticipated that not all persons volunteering to be part of the study would be assessed as eligible. It was also anticipated that not all persons volunteering to be part of the study would attend the baseline survey or those randomised to the J2SI Phase 2 program would engage.

### Eligibility and exclusion criteria

Participants are deemed eligible for J2SI program support (and the RCT) if they meet all the following criteria: (1) being aged 25 to 50 years at the time of enrolment (the age group that SHM serve in their homelessness programs); and (2) currently experiencing homelessness (i.e., sleeping rough, in temporary supported accommodation services, in a boarding or rooming house without tenure rights and private facilities, or couch-surfing), or housed for 6 months or less and at direct risk of homelessness due to having received an imminent eviction without a secure housing option available; and (3) having a history of chronic homelessness; and (4) being entitled to Australian welfare payments; and (5) not currently engaged in another long-term homelessness intensive support program. For the purposes of this study, chronic homelessness was defined by J2SI Phase 2 program administrators (i.e., SHM) as having a history of rough sleeping for 12 months continuously and/or at least three episodes of homelessness in the last 3 years.

Otherwise eligible participants are excluded if they meet any one of the following criteria: (1) not being fluent in English, such that an interpreter service would be required (budget constraints preclude interpreter service support); or (2) experiencing unmanaged severe mental illness that precluded provision of informed consent and/or would impede completion of surveys, even with a guardian present; or (3) being deemed by agency staff to pose a safety risk to staff, researchers or the participant themselves.

**Fig. 1:**
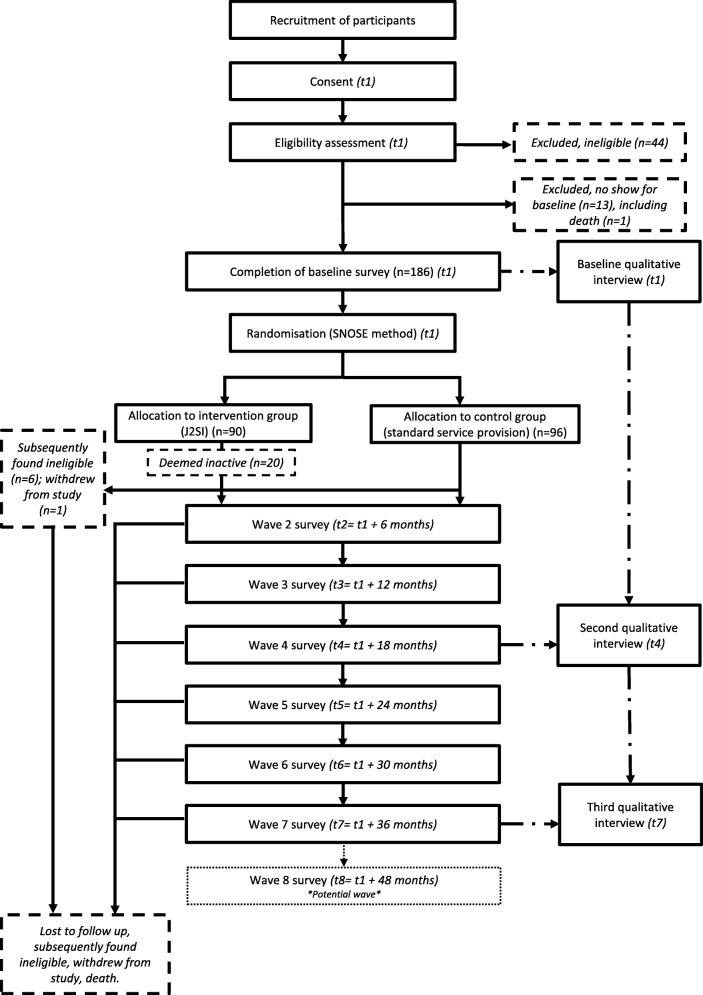
Overview of participant enrolment, randomisation and program evaluation. Note: t1 occurred between 07 January 2016 and 30 September 2016, depending on when the baseline survey was completed

Among those expressing interest to be involved in the study, 44 did not meet eligibility criteria and were excluded from the study prior to baseline. A further 13 participants were assessed as eligible for the study but did not attend the baseline survey interview, including one enrolee who died prior to the baseline survey (see Fig. 1). This resulted in a total of 186 persons who consented and completed the baseline survey. Post-randomisation, six individuals in the intervention group were excluded from the study as they were retrospectively deemed to not meet eligibility criteria by J2SI Phase 2 support staff, and one individual in the comparison group formally withdrew from the study.

### Randomisation

After written informed consent was obtained and the baseline survey completed, participants were randomly assigned into either the J2SI Phase 2 (intervention) group or the standard service provision (comparison) group. Although randomisation by computer-generated allocation [46, 47] is often the preferred method, SHM staff informed the research team that some participants in the J2SI pilot study had expressed concern that they felt computer-generated allocation was in some way manipulated to achieve a particular outcome. In response to their concerns, the researchers instead utilised the Sequentially Numbered, Opaque, Sealed Envelope (SNOSE) method [48]. This method is an effective and pragmatic method of randomisation that does not require specialised technology [48].

Due to the nature of the intervention, neither the participants, nor the J2SI Phase 2 support workers could feasibly be blinded to the allocation outcome. However, allocation to the treatment group has not been disclosed to third parties, including other support services.

### Participant retention

Efforts were made to establish trust and rapport with participants at the time of enrolment. In an effort to reduce loss to follow-up, participants were asked to provide contact information for themselves and for friends, relatives, and service providers who may know their whereabouts in the future. Permission was sought to enable researchers to follow up with all contact persons/services provided. Moreover, participant informed consent was obtained to allow local homelessness services and Australia’s social security agency to release current contact details to researchers during the RCT.

Individuals randomly allocated to the intervention group who do not engage with the J2SI Phase 2 program may be assigned “inactive status” in accordance with J2SI Phase 2 program guidelines (a decision made by J2SI Phase 2 staff, not the research team), but will remain enrolled in the study and continue to be followed for interviews. Examples of non-engagement include J2SI program staff being unable to ever locate or contact the participant following the baseline survey, relocation of participants outside the geographical scope of support, missing numerous agreed appointments, or failure to respond to regular contact attempts by intervention staff. As a result, the number of participants assessed for eligibility in the study was increased beyond the initial target of 60 per group to account for the rate of participants assigned “inactive status” by SHM during the baseline survey conduction period (*n* = 20).

Participants in the study continue to be followed up for both quantitative and qualitative interviews over a three-month period from the due date of an interview. Typically, 6–10 contacts attempts are made but in some cases more will be made. The participant will be deemed to have missed a particular wave if more than 90 days have passed from the date of the scheduled interview. However, participants will remain in the study, and contact attempts will be made at subsequent waves. All contacts are made by research assistants involved in the study who are responsible for conducting the interviews. It is expected that participants in the comparison group will be more difficult to follow up than those in the intervention and that follow-up rates may be lower than those recorded in the pilot study due to recruitment occurring across a larger geographic catchment zone that extends beyond the immediate area where SHM delivers services.

### Interventions

#### Intervention group: J2SI phase 2 program

The J2SI Phase 2 model draws on domestic and international research that demonstrates chronically homeless individuals benefit from individually tailored, intensive support [29, 49, 50]. Integral to the J2SI model is the provision of intensive, long-term (three years) case management and service coordination provided by case managers with low caseloads (1:6 worker-to-client ratio). Participants randomised to the J2SI Phase 2 intervention group receive intensive case management to address individual, interpersonal, and systematic barriers that are known to increase the difficulty of exiting chronic homelessness. The J2SI Phase 2 approach to case management is relationship-based, trauma-informed, and strengths-focussed. Non-therapeutic skill-building exercises, referrals to relevant services (e.g., vocational, employment, mental health), and tenancy support are provided. The overall aim is to deliver services that promote reintegration into mainstream society and lead to improved health and wellbeing through fostering competence in navigating support services, increasing social participation and developing capacity for independence, work, and sustained housing. The J2SI Phase 2 model also includes formal partnerships with public housing and community housing providers to increase access to housing opportunities and support a Housing First model of rapid transition of participants to permanent housing. As the J2SI Phase 2 program did not have guaranteed housing for all J2SI Phase 2 program participants prior to the start of the program, an integral component in the evaluation is measuring the extent to which the partnerships resulted in a significantly higher rate of access to housing and sustaining accessed housing over time.

#### Comparison group: standard service provision

Participants randomised to the ‘standard service provision’ or ‘services-as-usual’ group will potentially be able to access services provided by Melbourne homelessness services which typically involves some level of case management (but not the intensity and breadth of the J2SI Phase 2 program), possible referral to a range of services such as community-based mental health and drug and alcohol services, access to short-term crisis accommodation, and advocacy for permanent housing. No restrictions are placed on the comparison group participants in terms of utilisation of services and enrolment in non-J2SI Phase 2 intensive case management programs and housing opportunities that arise during the course of the J2SI Phase 2 program.

### Data collection

Three types of data will be collected: (1) quantitative, self-report survey data; (2) qualitative, semi-structured interviews; and (3) linked administrative data from public health services (e.g., medical records), specialist homelessness services, public housing, and the justice system (e.g., police and justice services records).

#### Quantitative measures

Self-report surveys will be administered in an interview format by research assistants at seven time-points during the three-year study (i.e., at baseline and every 6 months thereafter), with data entered into Qualtrics [51]. Research assistants will be trained by lead co-investigators (PF, MT) during a full-day training workshop and several follow-up sessions. Each research assistant will receive a manual that includes a detailed interview protocol and copies of survey instruments. During training sessions, research assistants will have the opportunity to practice administering the survey and to demonstrate decision-making skills in response to various participant presentations via hypothetical role-play scenarios.

Surveys collect data on socio-demographic characteristics, history of homelessness, behavioural problems, childhood exposure to family violence, time spent in out-of-home care, lifetime trauma history, current and past employment, justice system involvement, mental and physical health, substance use, health and support service utilisation, social networks, and overall quality of life. Where possible, validated instruments are utilised. Table 1 provides an overview of key instruments to be administered in each survey wave. Participants in both groups will be provided $40 cash (AUD) at each wave of data collection as reimbursement for their time.

**Table 1:** Domains and Variables Assessed Using Self-Report Measures at Each Time Point

Domains and Variables	Time Points							
	*t*1 (baseline)	*t*2	*t*3	*t*4	*t*5	*t*6^a^	*t*7^a^	*t*8^b^
Demographics								
General demographics *(*e.g.*, date of birth, gender, Aboriginality)*	X							
Education *(highest attainment and current participation)*	X	X	X	X	X	X	X	X
Homelessness and Housing								
Housing history *(6 months, 12 months, or lifetime [depending on wave])*	X	X	X	X	X	X	X	X
Current living arrangement *(last night/week)*	X	X	X	X	X	X	X	X
Adequacy of accommodation *(*e.g.*, safety, distance to services, affordability)*	X	X	X	X	X	X	X	X
Housing location and mobility					X	X	X	X
Life Experiences and Skills								
Independent Living Skills Scale – Homelessness [60]	X	X	X	X	X	X	X	X
Problems experienced *(*e.g.*, gambling, reading and writing)*	X	X	X	X	X	X	X	X
Family, Relations and Social Support Networks								
History of violence in the family home	X							
History of out-of-home care	X							
Relationship status	X	X	X	X	X	X	X	X
Children *(number, living arrangement, out-of-home care)*	X		X	X	X	X	X	X
Current contact with friends, family and social participation	X	X	X	X	X	X	X	X
Enriched Social Support Instrument (ESSI) [61]	X	X	X	X	X	X	X	X
Three Item Loneliness Scale (3-ILS) [62]	X	X	X	X	X	X	X	X
Support received from services	X	X	X	X	X	X	X	X
General Health								
Selected items from the 36-Item Short Form Survey (SF-36) [63];	X	X	X	X	X	X	X	X
Diagnoses of specified conditions and treatment	X	X	X		X	X	X	X
Health service utilisation *(*e.g.*, ED presentations, hospital admissions)*	X	X	X		X	X	X	X
Mental Health and Wellbeing								
Short Warwick-Edinburgh Mental Well-being Scale (S-WEMWBS) [64]	X	X	X	X	X	X	X	X
Kessler Psychological Distress Scale (K10) [65]	X	X	X	X	X	X	X	X
Depression Anxiety Stress Scales, Short Form (DASS21) [66]	X	X	X	X	X	X	X	X
Single-Item Self-Esteem Scale (SISES) [67]	X	X	X	X	X	X	X	X
Mental health diagnoses and treatment	X	X	X		X	X	X	X
Quality of Life								
World Health Organisation Quality of Life- BREF (WHOQoL-BREF) [68]	X	X	X	X	X	X	X	X
Trauma								
World Health Organisation Composite International Diagnostic Interview (WHO-CIDI; Trauma History Section) [69]				X				
Abbreviated PTSD Checklist Civilian Version (Abbreviated PCL-C) [70]	X	X	X	X	X	X	X	X
Alcohol and Drug Use								
Alcohol, Smoking and Substance Involvement Screening Test (ASSIST) [71];	X	X	X	X	X	X	X	X
Selected items from the Opiate Treatment Index (OTI) [72];	X	X	X	X	X	X	X	X
Use of alcohol and drug detox services	X	X	X		X	X	X	X
Economic Participation								
Labour force participation, employment (hours and occupation), unemployment, volunteering	X	X	X	X	X	X	X	X
Sources and level of income	X	X	X	X	X	X	X	X
Justice System								
Involvement with justice system *(*e.g.*, police, arrests, prison)*	X	X	X		X	X	X	X

^a^ Topics indicated in this table are proposed topics for relevant waves and are subject to change

^b^ proposed 48-month follow up of survey participants where funding permits

Survey modifications will be made following t1 for questions not requiring repetition (i.e., demographics and childhood family experiences). Additional modifications will be made at t4 to include a comprehensive trauma assessment in order to measure the association between experience of traumatic events and mid-trial housing, mental health, and psychosocial outcomes.

Surveys will be conducted in rooms at the three community-based partner agencies. Specific protocols have been developed to ensure interviewer safety or if a participant presents for interview under the influence of alcohol or other drugs (i.e., interviews will not proceed); expresses distress associated with survey questions or reports suicidal ideation or intent; or indicates intent to engage in, or to disclose, criminal activities of a serious nature.

#### Qualitative interviews

Using semi-structured interview schedules, qualitative data will be collected on three occasions during the 3-year study (i.e., baseline, 18 months, 36 months) and analysed inductively. For this component of the research, computer-generated randomisation will be utilised to identify 30 participants in the intervention and comparison groups to participate in qualitative interviews. Fifteen participants from each group will be selected in order to account for attrition and possible non-consent, with an aim of completing at least 10 interviews per wave. At each time point, a one-hour individual interview will be conducted by co-investigators who have experience with qualitative research methods.

The baseline qualitative interview will explore participants’ homelessness journey and current experiences related to the outcome variables of interest in this study. Prompts that elicit participants’ hopes or goals for the next year will also be utilised. During the second and third interview (i.e., 18 and 36 months), interviewers will elicit determinants of change, or lack of change, according to stated experiences and goals from the prior interview. Thus, prompts for the second and third interview will be tailored to the unique goals and challenges each participant disclosed during the prior interview. For intervention group participants, the perceived impact of the J2SI Phase 2 intervention on participant’s well-being and in facilitating or blocking goal attainment will be explored.

In addition to interviewing selected study participants, at each time point, intensive case managers and supervisors will be invited to participate in a two-hour focus group. They will be asked to reflect on their experiences delivering the J2SI Phase 2 intervention and how they perceive the intervention is impacting participants. Discussion prompts will be aided by displaying a poster showing the study’s key outcome variables.

All individual and group interviews will be audio recorded (with consent) and professionally transcribed. Immediately following the interview, the interviewer will summarise their impressions of the interview in a memo, which will be shared with the qualitative research team. For each interview, RCT participants (but not intervention staff) will be provided $40 cash (AUD) as reimbursement for their time.

#### Linked administrative data

With consent from study participants, administrative records will be obtained through formal State and Federal data linkage processes. Data will include health service utilisation (e.g., mental health service utilisation, ED presentations, alcohol and other drug treatment services, public hospital and psychiatric hospital admissions), engagement with the justice system (e.g., jail time, court cases, arrests), public housing stays, and use of government-funded homelessness services and government income support payments (see Table 2 for an overview of linked administrative databases to be accessed). Specific ethics processes (beyond university-based human research ethics approval) are required in order to obtain linkage data held by the Australian and Victorian Governments. A Human Research Ethics Committee (HREC) application will be made under Population Health Research Network and Victorian Government protocols to an approved HREC for the Victorian Centre for Data Linkage to undertake the data linkage process (https://www2.health.vic.gov.au/about/reporting-planning-data/the-centre-for-victorian-data-linkage). The study will seek linked administrative data for the three-year period prior to enrolment in the study, the three-year study period, and 3 years beyond completion of the RCT. Information gathered by intensive case managers who are delivering the J2SI Phase 2 program will also be analysed as part of the study.

**Table 2:** Summary of Linked Administrative Databases to be Accessed

Dataset name	Short description
Victorian Admitted Episodes Dataset (VAED)	VAED and VEMD contain information pertaining to health outcomes and health service utilisation, and will be used to assist in the estimation of the costs of homelessness and the change in these costs over time.
Victorian Emergency Minimum Dataset (VEMD)	
Client Relationship Information System for Service Providers (CRISSP)	To determine history of family and domestic violence, out-of-home care, and other family history throughout childhood.
Integrated Reports and Information System (IRIS)	To identify access to sexual assault and family violence services.
Housing Integrated Information Platform (HIIP)	To determine participants’ history with the Office of Housing, including public housing tenancies, and how well tenancies are going. For example, if respondents have strikes/notices against them and if they are paying rent on time.
Alcohol and Drug Information System (ADIS)	To understand participants’ alcohol and other drug use and access of treatment services.
Client Management Interface/ Operational Data Store (CMI/ODS)	To ascertain levels of access to different mental health services and to identify if there is a history of identified self-harm/suicide attempts.
Courts Services (Victoria) data	To determine the impact on housing interventions and interaction with the justice system. These databases will be used to assist in the estimation of the costs of homelessness and the change in these costs over time.
Department of Corrections (Victoria) data	
Victorian Police (VicPol) data	
Centrelink data	To ascertain changes in payments and other circumstances in participants’ lives generally and in conjunction with the intervention. Centrelink data is an alternative source to self-report data in a range of areas such as employment, income, Centrelink payment type, and location that we are collecting from participants.
Specialist Homelessness Services data collection (Victoria) from Specialist Homelessness Information Portal (SHIP)	SHIP data contains detailed client information that will provide insight into participants’ utilisation of homelessness services.
National Cause of Death Unit Record Files	The Causes of Death Unit Record Files provide information on the causes of death that occur and are registered in Australia.

### Data management and analyses

All study data and participant information will be stored in password-protected computer files at the UWA Centre for Social Impact, and at Swinburne University of Technology. Access to data will be limited to specific members of the research team. Participant data will not be released outside of the study, unless required by law.

#### Quantitative data analyses

Data will be analysed using SPSS 24 and StataMP 14 (or subsequent versions of these programs). For our primary analyses, we will utilise a modified intention-to-treat method to test study hypotheses using longitudinal survey data and linked administrative data, excluding only those participants who were randomised to the intervention group, completed a baseline survey, but then deemed to have not met the eligibility criteria by the intensive case management team (*n* = 6) or who subsequently withdrew consent (*n* = 1). Individuals randomised to the intervention group but deemed inactive at any time throughout the intervention will still be included in analyses. Intention-to-treat analyses will enable us to take into account the non-random nature of ‘ineligible’ and ‘inactive’ status.

Baseline characteristics of participants will be reported using descriptive statistics, including measures of central tendency and dispersion. Planned analysis includes a combination of statistical tests of difference between the treatment and control groups, and multivariable regression analyses (including random effects mixed modelling) will be used. In adjusted analyses, we will control for the effects of relevant demographic variables, covariates known to be strong predictors of outcomes (e.g., duration of prior homelessness), and variables that reflect any group differences at baseline. We will examine associations between key outcome variables and relevant demographic variables (e.g., age, gender), as well as various aspects of participant trauma history (e.g., type of trauma experienced).

#### Qualitative data analyses

Participant transcripts will be uploaded to Quirkos© qualitative data analysis software, then independently interpreted and coded by members of the qualitative research team who are from, or affiliated with Swinburne University of Technology. To ensure accurate transcription, members of the qualitative team will review the transcription while listening to the audio recording prior to data analysis. This will ensure a more accurate understanding of the data via the gathering of contextual information, such as tone and affect, while confirming accuracy of transcription [52, 53]. Themes from SHM intervention staff focus groups will be shared with participating staff to ensure data quality and confirm agreement with interpretive decisions. For study participant interviews, and to ensure consistency in coding, the first ten control group interviews will be coded by all qualitative researchers. Subsequently, a double coding process will be undertaken in which two members of the qualitative research team will independently code each interview transcript. Discrepancies in coding will be scrutinised and resolved by the researchers. A description of how and why analytic decisions were made will be recorded throughout the data analysis period [54, 55]. A line-by-line coding technique will be employed to establish themes and sub-themes within and across interviews and an evolving codebook will be created, as recommended by Crabtree and Miller [56].

#### Integration of qualitative and quantitative data

Consistent with a convergent design [57], and at analysis stage, specified research questions will be addressed in three ways. Firstly, data derived from focus groups with SHM intervention staff and in-depth individual interviews with homeless participants will be triangulated to enable researchers to gain a deeper understanding of the impacts of the J2SI intervention and to explore how the perspectives of staff and recipients align or differ in the J2SI intervention [58]. Secondly, within the constraints of the small qualitative sample (*n* = 20), we will synthesise both qualitative and quantitative data to investigate within, and between group phenomena. For instance, groups of participants may be sorted for more in-depth analysis or for comparison purposes (e.g., those with early onset homelessness versus late onset homelessness). Lastly, qualitative and quantitative data will be integrated and analysed across time points to comprehensively describe participant experiences in the intervention and comparison groups, respectively.

#### Program governance and ethics

SHM established a J2SI Phase 2 program steering committee as well as an evaluation sub-committee prior to the start of the research study. The J2SI Phase 2 program steering committee is independently chaired. It is comprised of representatives from SHM, program funders, SHM board members, service providers engaged in the program, state government and the lead Chief Investigator of the research team. The steering committee and evaluation sub-committee receive briefings from the research team on the development of the research who in turn receive advice on the study from committee members.

The research study received Human Research Ethics Committee approval from UWA and Swinburne University. The full study protocol (version 1) is fully available to the public via Australian New Zealand Clinical Trials Registry (see Additional file 1: Table S1 for further information).

#### Economic evaluation

An economic evaluation will examine two key issues. Firstly, it will investigate whether a significant difference is found between the intervention group and the comparison group in terms of the costs of homelessness over time (in particular health and justice costs). It has been well documented that individuals experiencing chronic homelessness frequently access high-cost hospital-based services, rather than lower-cost general practitioner or allied health services. Conversely, when people are housed and supported in their tenancy, use of tertiary services decreases and access to primary care increases [32, 34, 38–40]. However, there can be an initial increase in tertiary health care utilisation, as previously untreated health conditions are addressed [33]. For example, where individuals have previously undiagnosed mental health or other chronic health issues that continue to deteriorate while rough sleeping, being housed and supported by a caseworker provides a conduit to access services for ongoing assistance to manage their health. This, in-turn, may result in an increase in healthcare costs.

Costs will be assessed using both self-report data and linked administrative data on health service utilisation and engagement with the justice system. Secondly, the economic evaluation will examine the cost-effectiveness of the J2SI Phase 2 program. This will be estimated by comparing the differential outcomes achieved under the two alternatives to the additional cost of providing the J2SI Phase 2 intervention (relative to the cost of providing standard homelessness support services).

## Discussion

Given the high number of individuals currently experiencing chronic homelessness in Australia, the J2SI program provides a novel approach that allows more people to be supported to exit long-term homelessness. This study addresses an important gap in the literature on the impact of intensive case management interventions aimed at improving housing, health and psychosocial-related outcomes for chronically homeless people using an RCT design.

A strength of this study is the mixed methods RCT design which has not often been feasible in homelessness research to date. It will draw on rich longitudinal survey and qualitative interview data, together with extensive linked administrative data across a broad range of health, health service use, housing and justice system data. RCTs are gold standard research designs in the medical and healthcare arena but have been utilised less frequently in the community domain, even where programs have significant health and healthcare implications, as is the case with the J2SI Phase 2 program. This evaluation demonstrates that even in a constrained resource environment, it is feasible to conduct an RCT.

It is important to acknowledge the limitations of this study. Firstly, the study design does not include a double blind approach; both participants and researchers are made aware of the outcomes of the randomisation process. In social programs of this kind, however, it would not be feasible to provide the J2SI Phase 2 program without the participants knowing that they were receiving the treatment (given their knowledge of standard care) and the information provided on the program in the recruitment stage. Moreover, the research team was independent of the J2SI Phase 2 program, in that it was not engaged in the development of the program, nor in the provision of care, which were both the responsibility of SHM and its service providers. Second, individuals recruited for participation had a history of chronic homelessness; thus, these findings may not generalise to adults with transient periods of homelessness. Third, self-report measures may be inaccurate due to memory error, nondisclosure, social desirability or intentional misrepresentation [35]. However, to reduce the incidence of data inaccuracies, standardised tools validated for use with vulnerable populations were used where possible [59]. Moreover, we will obtain linked administrative data, which will enable us to examine the level of agreement between self-report responses and administrative data on some variables (e.g., ED presentations, hospital admissions). Lastly, this study will be conducted in a large, urban setting in Australia and findings may not generalise to smaller cities or to cities in other nations, particularly where social service systems differ substantially.

If the RCT discovers that the J2SI Phase 2 intervention demonstrates effectiveness in improving participant outcomes, it may be considered for broader national and international dissemination as an evidence-based intervention for chronically homeless adults.

## Additional file

Additional file 1: **Table S1.** Trial Registration Data. All details pertaining to the Australian New Zealand Clinical Trial Registry are provided in this file. (DOCX 23 kb)

## Abbreviations

ED: Emergency department
J2SI Phase 2: Journey to social inclusion phase 2
RCT: Randomised controlled trial
SHM: Sacred Heart Mission
SNOSE: Sequentially Numbered, Opaque, Sealed Envelope
UWA: The University of Western Australia
