# Supplementary material for: A mixed methods randomised control trial to evaluate the effectiveness of the journey to social inclusion – phase 2 intervention for chronically homeless adults: study protocol
Source: BMC Public Health. 2019 Mar 22;19:334. doi: 10.1186/s12889-019-6644-1 (PMC6431014; doi:10.1186/s12889-019-6644-1)
Supplement: Supplementary file 1 — Table S1. Trial Registration Data. All details pertaining to the Australian New Zealand Clinical Trial Registry are provided in this file. (DOCX 23 kb) [file 12889_2019_6644_MOESM1_ESM.docx]

Additional file 1: **Table S1.** Trial Registration Data

| Data category | Information |
| --- | --- |
| Primary registry and trial identifying number | Australian New Zealand Clinical Trials Registry  ACTRN12616000162415 |
| Date of registration in primary registry | 10 February 2016 |
| Secondary identifying numbers | Nil known |
| Source(s) of monetary or material support | Sacred Heart Mission |
| Primary sponsor | University of Western Australia |
| Secondary sponsor(s) | Swinburne University of Technology, Sacred Heart Mission |
| Contact for public queries | PF, MT |
| Contact for scientific queries | PF, MT |
| Public title | JOURNEY TO SOCIAL INCLUSION MARK II: A randomised control trial to assess a modified homelessness intervention (J2SI Mark II) for chronically homeless adults in Melbourne |
| Scientific title | JOURNEY TO SOCIAL INCLUSION MARK II: A randomised control trial to assess a modified homelessness intervention (J2SI Mark II) on mental health & wellbeing, social & economic participation and housing stability in chronically homeless adults in Melbourne |
| Countries of recruitment | Australia |
| Health condition(s) or problem(s) studied | Chronic homelessness |
| Intervention(s) | Intervention group: will receive support for up to three years through the J2SI program (intensive case management and service coordination; tenancy support and capacity building; trauma informed practice; skills for inclusions; fostering independence). |
|  | Comparison group: treatment as usual; individuals will not be prevented from accessing services and will not be impacted on their current level of support. However, will not receive intensive individualised support under the J2SI intervention. |
| Key inclusion and exclusion criteria | Ages eligible for study: 25-50 years Sexes eligible for study: both Accepts healthy volunteers: no |
|  | Inclusion criteria: (1) Have been sleeping rough continuously for more than a year OR who are homeless and have experienced several episodes of homelessness over the past three years OR who are currently permanently housed, are at risk of homelessness and have been homeless at some point in the last six months; AND (2) Have received some level of case management response from Sacred Heart Misson, VincentCare, St Marys House of Welcome OR are well engaged with an on-site Program but have been unable to secure a case management response; AND (3) Are aged between 25 – 50 years; AND (4) Are a permanent resident; AND (5) Are eligible to access public and/or community housing; AND (6) Agree to participate in the program. |
|  | Exclusion criteria: (1) People who are currently receiving other long term intensive support (e.g., Street2Home); OR (2) People experiencing language barriers that require an interpreter service, as budget constraints limit the ability to provide interpreter service support; OR (3) People experiencing an unmanaged mental illness of a severe nature affecting an ability to provide consent and complete a survey even with a guardian present; OR (4) People who for any reason are unable to (a) give informed consent or (b) participate fully in the intervention or study even with guardian present; OR (5) People deemed by agency staff to pose an identifiable safety risk to agency staff, researchers, other people or the participant themselves. |
| Study type | Type of study: Lifestyle intervention |
|  | Allocation: randomised intervention model (SNOSE method) |
|  | Masking: Blinded, masking used (outcomes assessor, investigators) |
|  | Assignment: Parallel |
|  | Purpose: Treatment |
|  | Phase: not applicable |
| Date of first enrolment | 8 January 2016 |
| Sample size | Anticipated: 130 |
|  | Actual: 186 |
| Recruitment status | Active, not recruiting |
| Primary outcome(s) | Outcome: Sustained permanent housing (ability to obtain and maintain a housing tenancy)  Metric/method: linked administrative data from the housing/public housing tenants database (the HiiP database)  *Timepoint: At baseline, then 6, 12, 18, 24, 30 and 36 months after baseline (7 time points in total). A further 48 month survey will be undertaken if funding available.* |
|  | Outcome: Changes in mental health & wellbeing  Metric/method: DASS21, S-WEMWBS, K10, SISES and linked administrative data from mental health operational data store (ODS), Victorian Admitted Episodes Dataset (VAED) and Victorian Emergency Minimum Dataset (VEMD).  *Timepoint: At baseline, then 6, 12, 18, 24, 30 and 36 months after baseline (7 time points in total). A further 48 month survey will be undertaken if funding available* |
|  | Outcome: Change in social participation  Metric/method: ESSI, 3-item loneliness scale, and self-report data on friends, family and community connections and participation.  *Timepoint: At baseline, then 6, 12, 18, 24, 30 and 36 months after baseline (7 time points in total). A further 48 month survey will be undertaken if funding available.* |
|  | Outcome: Change in capacity for independence  Metric/method: 'independent living skills-homelessness' an assessment tool designed specifically for this study.  *Timepoint: At baseline, then 6, 12, 18, 24, 30 and 36 months after baseline (7 time points in total). A further 48 month survey will be undertaken if funding available.* |
| Key secondary outcomes | Outcome: Change in economic participation  Metric/method: self-report and linked administrative data on employment status and income earned.  *Timepoint: At baseline, then 6, 12, 18, 24, 30 and 36 months after baseline (7 time points in total). A further 48 month survey will be undertaken if funding available.* |
|  | Outcome: Change in health outcomes  Metric/method: self report data and linked administrative data from VAED and VEMD  *Timepoint: Annual analysis of linked data (but also retrospectively looking at data prior to program entry). A further 48 month survey will be undertaken if funding available.* |
|  | Outcome: Compare the cost effectiveness of the program against treatment as usual in relation to service usage, emergency admissions, and contact with justice services  Metric/method: linked administrative data from VAED and VEMD  *Timepoint: Annual analysis of linked data (but also retrospectively examining data prior to program entry).* |
|  | Outcome: Change in health service usage  Metric/method: self report data and linked administrative data from VAED and VEMD  *Timepoint: Annual analysis of linked data (but also retrospectively looking at data prior to program entry)* |
| Ethics review | Status: Approved (RA/4/1/7904) |
|  | Date of Approval: 8 December 2015 |
|  | Contact details: HREC University of Western Australia, 35 Stirling Hwy, Crawley WA 6009 |
| Completion date | 30 September 2019 (or 30 September 2020 if funding available for additional wave of data). |
| Summary results | Results will be provided upon completion of the project. Reports will be made available via the Sacred Heart Mission website <https://www.sacredheartmission.org/understanding-homelessness/homelessness-projects-reports> |
| IPD sharing statement | No individual clinical trial participant-level data will be shared. |
